# Physiological and Transcriptional Analyses Reveal Differential Phytohormone Responses to Boron Deficiency in *Brassica napus* Genotypes

**DOI:** 10.3389/fpls.2016.00221

**Published:** 2016-02-26

**Authors:** Ting Zhou, Yingpeng Hua, Yupu Huang, Guangda Ding, Lei Shi, Fangsen Xu

**Affiliations:** National Key Laboratory of Crop Genetic Improvement, Microelement Research Center, Huazhong Agricultural UniversityWuhan, China

**Keywords:** ABA, boron deficiency response, *Brassica napus*, gene expression profiling, IAA, JAs

## Abstract

Phytohormones play pivotal roles in the response of plants to various biotic and abiotic stresses. Boron (B) is an essential microelement for plants, and *Brassica napus* (*B. napus*) is hypersensitive to B deficiency. However, how auxin responds to B deficiency remained a dilemma for many years and little is known about how other phytohormones respond to B deficiency. The identification of B-efficient/inefficient *B. napus* indicates that breeding might overcome these constraints in the agriculture production. Here, we seek to identify phytohormone-related processes underlying B-deficiency tolerance in *B. napus* at the physiological and gene expression levels. Our study indicated low-B reduced indole-3-acetic acid (IAA) concentration in both the shoots and roots of *B. napus*, and affected the expression of the auxin biosynthesis gene *BnNIT1* and the efflux gene *BnPIN1* in a time-dependent manner. Low-B increased the jasmonates (JAs) and abscisic acid (ABA) concentrations and induced the expression of the ABA biosynthesis gene *BnNCED3* and the ABA sensor gene *BnPYL4* in the shoot. In two contrasting genotypes, the auxin concentration decreased more drastically in the B-inefficient genotype ‘W10,’ and together the expression of *BnNIT1* and *BnPIN1* also decreased more significantly in ‘W10’ under long-term B deficiency. While the JAs concentration was considerably higher in this genotype, and the ABA concentration was induced in ‘W10’ compared with the B-efficient genotype ‘QY10.’ Digital gene expression (DGE) profiling confirmed the differential expression of the phytohormone-related genes, indicating more other phyohormone differences involving in gene regulation between ‘QY10’ and ‘W10’ under low-B stress. Additionally, the activity of DR5:GFP was reduced in the root under low-B in *Arabidopsis*, and the application of exogenous IAA could partly restore the B-defective phenotype in ‘W10.’ Overall, our data suggested that low-B disturbed phytohormone homeostasis in *B. napus*, which originated from the change of transcriptional regulation of phytohormones-related genes, and the differences between genotypes may partly account for their difference in tolerance (B-efficiency) to low-B.

## Introduction

A change in phytohormones is one of the most significantly adaptive strategies used by plants in their response to adverse conditions (Potters et al., 2007). Recently, the roles that phytohormones play in response to limited or excessive nutrients (including nitrogen, phosphorus, potassium, sulfur, and many others) have been well-characterized (Scheible et al., 2004; Liu et al., 2009; Rubio et al., 2009; Kapulnik et al., 2011; Kiba et al., 2011; Romera et al., 2011; Iqbal et al., 2013; Zhang et al., 2014). On one hand, plant mineral nutrition status affects phytohormone metabolism, and interaction. Nitrogen regulated cytokinin (CTK) biosynthesis by triggering the expression of CTK biosynthesis-related genes *IPT3* and *CYP735A* (Kiba et al., 2011) and also facilitated cell-to-cell auxin transport by regulating the abundance of NRT1.1/CHL1 (Ho et al., 2009), known as a dual affinity nitrate transporter and nitrate sensor (Krouk et al., 2010). A low potassium supply can induce the increase in the expression of ethylene (ETH) biosynthesis-related genes, thus boosting up ETH concentration, which was closely correlated with the phenotype of reduced lateral roots when *Arabidopsis* was exposed to potassium deficiency (Shin and Schachtman, 2004; Jung et al., 2009). *OsARF16* was involved in the crosstalk of auxin signaling and iron deficiency signaling transduction pathway (Shen et al., 2015). The genes involved in the first three steps of the jasmonates (JAs) biosynthesis enzymes, i.e., LOX2, AOS, and AOC, were significantly up-regulated in *Arabidopsis* during potassium starvation and down-regulated after potassium resupply (Armengaud et al., 2004). The JAs metabolism pathway was considered as one of the most significant pathways identified in rice under arsenic stress, although arsenic is not an essential element (Yu et al., 2012). On the other hand, spraying exogenous phytohormones or knockdown of phytohormone biosynthesis/signaling-related genes could alter the expression of some genes implicated in nutrient uptake and transport, which may, to a certain extent, lead to the restoration of phenotypes caused by unfavorable nutrient conditions. It was reported that CTK repressed the phosphate-starvation response through increasing the intracellular phosphate level (Wang et al., 2006). Auxin treatment inhibited the expression of a low-sulfur-responsive gene in *Arabidopsis* (Dan et al., 2007). Treating maize plants with auxin increased the gene expression of a potassium transporter ZMK1 (Philippar et al., 1999). *Arabidopsis* mutants, *abi2-1*, and *aba1*, which showed low sensitivity or impaired abscisic acid (ABA) biosynthesis, exhibited a reduced accumulation of anthocyanin when grown under low-Pi (inorganic phosphate), whereas not all of the plant defects caused by low-Pi were able to be rescued (Trull et al., 1997; Ciereszko and Kleczkowski, 2002). Exogenous ABA alleviated zinc uptake and accumulation when *Populus* was exposed to excess zinc (Shi et al., 2015).

Boron (B) deficiency is universally acknowledged as a serious agricultural problem worldwide, especially in Japan, China, USA, and Brazil (Tanaka and Fujiwara, 2008). To cope with B deficiency, plants trigger physiological and developmental responses, aiming to strengthen tolerance to B deficiency, which in many cases alters morphology and metabolism and involves changes in phytohormones. A dramatic decrease in IAA exporting from the shoot apex as well as a decline in the IAA concentration in the shoot apex and roots of pea plants have been observed (Li et al., 2001; Wang et al., 2006). Nonetheless, another viewpoint supported that excessive auxin accumulated in bean radicles (Coke and Whittington, 1968), and a similar result was observed in lettuce (Crisp et al., 1976). Under a low-B condition, the auxin efflux gene *PIN1* (pin-formed 1) was down-regulated and *PIN2* (pin-formed 2) played an important role in the root meristem maintenance in *Arabidopsis* (Li et al., 2015). The use of *Arabidopsis* IAA and ETH mutants has provided substantial evidence, which suggested that IAA and ETH were involved in the inhibition of root elongation, and the formation and elongation of root hairs under B deficiency, respectively (Martin-Rejano et al., 2011). Abreu et al. (2014) formulated the hypothesis that signaling mechanisms during cell differentiation and organogenesis were extraordinarily sensitive to B deficiency in the apical root meristem of *Arabidopsis*, and CTK functioned as the key regulator. B deficiency inhibits root cell elongation and this response is driven by a signaling pathway with the participation of ETH and auxin (Camacho-Cristobal et al., 2015). Using cDNA chips, several differentially expressed genes (DEGs) related to phytohormones were identified in *Arabidopsis* under B deficiency, including JAs, CTK, IAA, ETH, and ABA (Peng et al., 2012). Although the relationships between B deficiency and phytohormone responses have been reported in numerous studies, the actual auxin response to B deficiency remained a dilemma for many years and how other phytohormones respond to B deficiency remain elusive. In addition, an overall analysis is rare on transcriptional regulation for how phytohormones respond to B deficiency.

*Brassica napus* (*B. napus*), in addition to being one of the preeminent sources of cooking oil worldwide (Meyer, 2009), is an agricultural crop that is highly susceptible to B deficiency (Marschner, 1995). The B-efficient (B-deficiency tolerant) genotype ‘QY10’ was identified to cope well with low-B conditions compared with the B-inefficient (B-deficiency sensitive) genotype ‘W10’ (Xu et al., 2002; Zhao et al., 2008). identification of Differential protein abundance and cDNA microarray analysis showed that JAs-, CTK-, IAA-, ETH-, ABA-related genes, and JAs-related proteins were characterized under B deficiency (Wang et al., 2011; Peng et al., 2012), suggesting that *B. napus* may have evolved sophisticated mechanisms involving changes in phytohormones to enhance the ability to tolerate B deficiency. The physiological variation in the sensitivity of different *B. napus* genotypes to low-B highlights the possible complex architecture of plant phytohormone responses. The relationship between B and auxin has not yet been elucidated and there is little information on the relationship between B and other phytohormones. Consequently, studies on phytohomone-mediated response to low-B or the effect of low-B on phytohormones and related gene expression in *B. napus* are crucial for in-depth researches determining the differences in phytohormone responses between B-efficient and B-inefficient genotypes. In this study, we hypothesized: (i) that B deficiency could induce the common and/or distinct phytohormone responses, including concentration and transcriptional regulation levels between B-efficient and B-inefficient *B. napus* genotypes; and (ii) that the distinct phytohormone responses would be one reason for the severity differences in response to B deficiency between B-efficient and B-inefficient *B. napus* genotypes. The objectives of this study were to elucidate the changes in *B. napus* phytohormones in response to B deficiency, at both physiological and molecular levels, as well as the differences in phytohormone responses between B-efficient and B-inefficient *B. napus* genotypes.

## Materials and methods

### Plant materials and growth condition

Two different *B. napus* genotypes, including B-efficient ‘QY10’ and B-inefficient ‘W10’ (Xu et al., 2002; Zhao et al., 2008), were used to conduct two different B deficiency hydroponic experiments. Plump seeds from the two genotypes were surface-sterilized using 0.5% (w/v) NaClO for 10 min and were rinsed completely in ultrapure water (18.25 MΩ·cm). The seeds were then sown in gauze after being soaked in deionized water for 24 h. After 5 days for germination, uniform seedlings were transplanted into black plastic containers with Hoagland and Arnon solution (Hoagland and Arnon, 1950). Under long-term exposure to low-B, seedlings were first grown in one-quarter-strength nutrient solution containing low-B (0.25 μM H_3_BO_3_), and sufficient-B (25 μM H_3_BO_3_) was used as control. B concentration was determined according to Asad et al. (1997) and Zhao et al. (2008). The nutrient solution was replaced every 5 days, beginning with one quarter-strength solution, afterwards progressing to one-half-strength and eventually full-strength. The leaves, shoot apexes, root crowns, and roots were harvested separately on the 20th day. When exposed to short-term B-free starvation treatment, seedlings were first grown under sufficient-B condition (10 μM H_3_BO_3_) for 12 days, then the roots of half plants were repeatedly rinsed with ultrapure water to remove B on the surface and transferred to B-free solution afterwards, and the rest seedlings transferred to sufficient-B circumstance (10 μM H_3_BO_3_) as control. The leaves, shoot apexes and roots were harvested separately at the 0, 3, 6, 12, 24, 72, and 120 h after transferred to B-free condition. For digital gene expression (DGE) profiling experiment, seedlings were grown under low-B (0.25 μM) condition for 10 days when ‘W10’ showed slight B-defective symptoms, then the leaves and roots of ‘W10’ and ‘QY10’ were harvested. All plants were cultivated in an illuminated culture room with a temperature regime of 24/22°C (day/night) and a photoperiod of 14/10 h (day/night) and a light intensity of 300–320 μmol·m^−2^·s^−1^. Each treatment was set with four replicates. For the exogenous IAA application experiment, plants were grown under sufficient-B or low-B condition for 10 days, then transferred to either 0.25 or 25 μM B with either 0 or 10 μM IAA (Sigma-Aldrich) for another 10 days, then four plants from each group were harvested. *Arabidopsis* harboring DR5:GFP (a synthetic auxin-inducible promoter-drived green fluorescent protein) and PIN1::PIN1-GFP seeds were kindly provided by Hongwei Xue and Yingtang Lu, respectively. *Arabidopsis* plants were grown in MS medium under sufficient-B (30 μM) or low-B condition (0.1 μM) under standard greenhouse conditions with artificial light (16/8-h photoperiod) for 4 or 12 days. The roots were harvested carefully for microscopy analysis.

### Determination of root architecture

Plant roots were imaged using a scanner after exposure to low-B for 20 days. Total root length, root volume and root surface area were measured using the root image analysis software Win-RHIZO Pro (Regent Instruments, QC, Canada).

### Quantification of B concentration and B efficiency coefficient

Samples were prepared from four plants for each replicate. All samples were killed out at 105°C for 30 min, and then oven-dried to constant weight at 65°C. The dry samples were then weighed and ground to fine powder in a carnelian mortar. B was extracted from the dry powder by shaking it in 10 mL of 1 M HCl for shoot and 6 mL for root in a shaker for 2 h. The B concentration was determined by Inductively Coupled Plasma-Optical Emission Spectroscopy (ICP-OES, Varian, Inc. USA). Plant dry matter (DM), B concentration, B content, and B efficiency coefficient (BEC = DM under B deficiency/DM under sufficient B) were calculated for each genotype, respectively.

### Digital gene expression (DGE) library preparation and sequencing

Leaves and roots of the seedlings with three biological replicates were individually sampled. Total RNA was extracted with Trizol Reagent (Invitrogen, CA, USA) from the young leaves and roots of the two genotypes ‘QY10’ and ‘W10.’ Reverse transcriptions were carried out using M-MLV Reverse Transcriptase (Promega, WI, USA) according to the manufacturer's instructions. Tag libraries from the RNA samples were prepared in parallel, and then were subjected to an Illumina Hiseq 2500 platform, generating 50 bp single-end reads. For differentially expressed genes (DEGs) analysis, the number of expressed tags was calculated and then normalized to FPKM (fragment per kilo base of exon per million fragments mapped) using Cuffdiff 2.0, a software known to robustly perform differential analysis (Transcript assembly and quantification by RNA-Seq reveals unannotated transcripts and isoform switching during cell differentiation; significance level at *P* < 0.05, false discovery rate [FDR] < 0.05).

### JA, ABA, and IAA analysis

Samples were prepared using a slightly modified crude extraction procedure reported by Liu et al. (2012). The standard IAA, JA were purchased from Sigma-Aldrich (St. Louis, MO, USA), and ABA, OPDA (JA biosynthetic precursor 12-oxophytodienoic) and JA-Ile (the isoleucine conjugate of JA) were from OlChemIm (OlChemIm, Olomouc, Czech Republic). The internal standards were ^2^H_6_ABA (OlChemIm) for ABA, 10-dihydro-JA (DHJA; OlChemIm) for JA, OPDA and JA-Ile, and D_2_-IAA (Sigma-Aldrich) for IAA. The modified steps were using internal standards, 30 ng ^2^H_6_ABA, 40 ng DHJA, and 50 ng D_2_-IAA for each sample, and the filtrate was dried then dissolved in 200 μL 50% methanol. Total IAA, JA, OPDA, JA-Ile, and ABA were determined by ultra-fast liquid chromatography-electrospray ionization tandem mass spectrometry (UFLC-ESI-MS).

### Quantitative real time PCR

Total RNA was extracted and reversely transcribed as above in digital gene expression (DGE) library preparation and sequencing method. Gene sequences were achieved from the available *B. napus* genome sequence database (http://www.genoscope.cns.fr/brassicanapus/) and degenerate primers were designed by Primer Premier 5.0. Quantitative real time polymerase chain reaction (qRT-PCR) for detecting the relative expression of genes was performed using the SYBR Green Real-Time PCR Master Mix Kit (TOYOBO, Japan) and the CFX96^TM^ Real-Time PCR Detection System (Bio-Rad, Hercules, CA, USA). All PCR reactions were performed with independently three replicates. The PCR profiles were as follows: 95°C for 1 min, followed by 45 cycles of 95°C for 10 s, 60°C for 15 s, and 72°C for 15 s. The sequences of primers used for qRT-PCR were listed in Supplementary Table 1.

### Microscopy

Images of plant roots were observed with a light/fluorescence microscope (Nikon 80I). Root tips of 12-day *Arabidopsis* DR5:GFP and PIN1::PIN1-GFP were mounted onto microscope slides for observation. Confocal microscopy was performed using a Leica Sp2 Confocal Laser-Scanning Microscope according to the manufacturer's instructions. The 4-day DR5: GFP roots were mounted onto microscope slides with 20 μg mL^−1^ propidium iodide (PI, Sigma-Aldrich). Ten seedlings were analyzed per treatment. The signal intensity was measured using ImageJ software (W.S. Rasband, National Institutes of Health), and error bars were obtained based on measurements of 10 seedlings per treatment.

### Statistical analysis

Analysis of variance was conducted by SPSS16.0 for Windows software (SPSS 249 Inc, Chicago, IL, USA). The long-term and short-term experiments were conducted separately and the data were dealt separately. Differences among the B-treatments were identified using the least significant difference test (LSD) at the *P* < 0.05 level. Expression data were normalized with the expression level of the actin by the 2$2^{-\Delta \Delta C}{}_{\text{T}}$ method (Livak and Schmittgen, 2001).

## Results

### Variation of two genotypes in response to low-B stress

**Figure 1:**
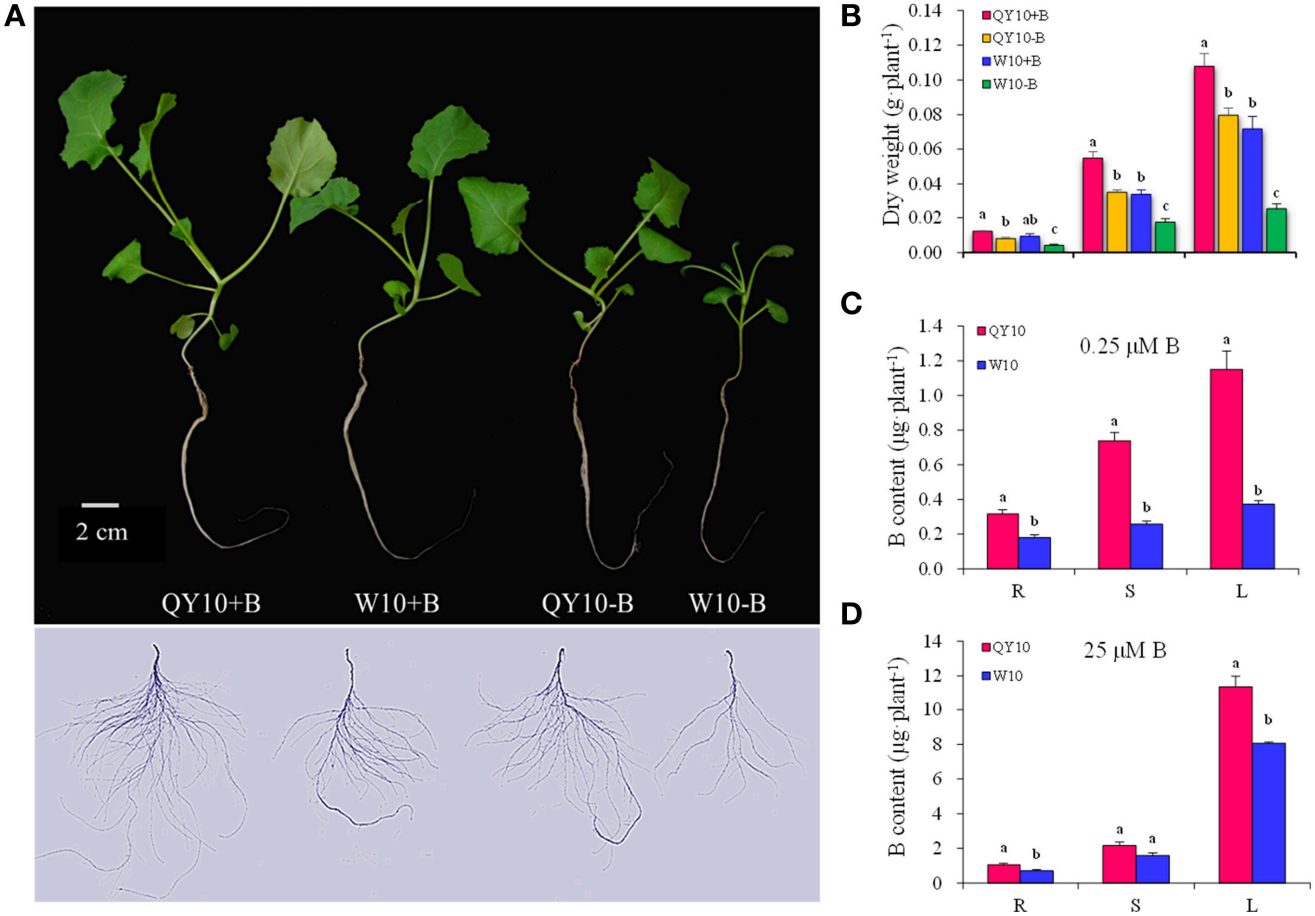
**Growth variations of ‘QY10’ and ‘W10.’** **(A)** Phenotype, **(B)** dry weight, **(C, D)** B content of two *B. napus* cultivars ‘QY10’ (B-efficient) and ‘W10’ (B-inefficient) grown hydroponically under sufficient B (+B, 25 μM B) or low-B (−B, 0.25 μM B) for 20 days. R, root; S, stem; L, leaves. Values represent mean ± SD of four independent replicates, and bars with different letters show significant differences for the same organ among the treatments (ANOVA, LSD, *p* < 0.05).

The biomass (dry weight), B content, and B efficiency coefficient (BEC) of two *B. napus* genotypes, i.e., B-efficient ‘QY10’ and B-inefficient ‘W10,’ were measured and analyzed at the tested B treatments. Under the low-B (0.25 μM) condition, ‘W10’ exhibited typical B-defective symptoms, including curved and thickened leaves, pluriceps shoot apex, short primary root and hypogenetic lateral roots (Figure 1A). In addition, the dry weight of the leaves, stem and root and the B content of ‘W10’ were significantly lower than those of ‘QY10’ (Figures 1B,C). In the presence of sufficient B (25 μM) for 20 days, ‘QY10’ slightly outperformed ‘W10’ (Figure 1A) and accumulated slightly more B (Figure 1D). The BEC of ‘QY10’ reached 0.70, which was markedly higher than that of ‘W10’ (0.40). Analysis of the total root length, root surface area and root volume of the two *B. napus* genotypes confirmed that ‘W10’ was more sensitive to B deficiency than that of ‘QY10’ (Supplementary Figure 1). These results demonstrated that ‘QY10’ has a greater tolerance (higher B efficiency) to low-B stress than ‘W10.’

### Digital gene expression (DGE) profiling of phytohormone-related genes

**Figure 2:**
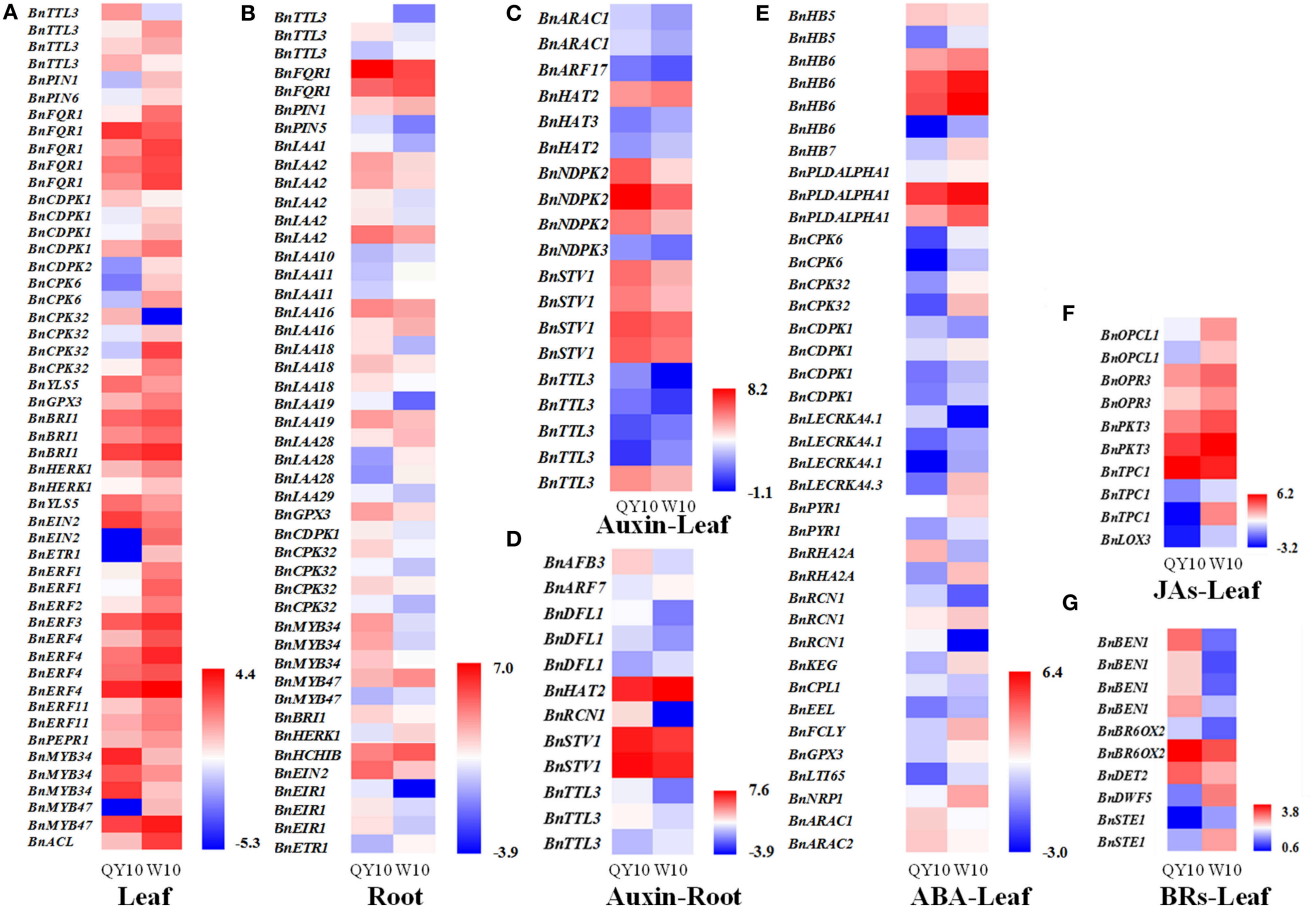
**Comparative analysis of phytohormone-related gene expression profiling of ‘QY10’ and ‘W10’ in response to low-B stress**. **(A,B)** Heat map representing the phytohormone-related DEGs responding to stimuli and the transcription factors Aux/IAAs, MYBs, and ERFs, which were differentially expressed in the leaves **(A)** and root **(B)** between ‘QY10’ and ‘W10’ under low-B stress. The color from blue to red represents log_2_ FPKM (Fragments Per Kilobases of exon model per Million mapped reads). **(C–G)** Heat map representing the DEGs involved in the auxin-mediated signaling pathway in the root **(C)** and leaves **(D)**, ABA-mediated signaling pathway in the leaves **(E)**, JAs biosynthetic process in the leaves **(F)**, and BRs biosynthetic process in the leaves **(G)** of ‘QY10’ and ‘W10.’

To assess a comprehensive characterization of different phytohormone transcriptional behaviors under B deficiency, DGE analysis was performed on both the root and leaves of ‘QY10’ and ‘W10.’ Global expression profiling of the genes related to eight phytohormones, including auxin, ABA, ETH, brassinosteroids (BRs), JAs, Gibberellin (GA), CTK and salicylic acid (SA), was conducted. The results showed that among 2,744 phytohormone-related genes, 512 in the root, and 728 in the leaves were indentified to be differentially expressed between ‘QY10’ and ‘W10’ under a 10-day B-deficiency period, accounting for 18.7 and 26.5% of the total, respectively (Supplementary Table 2). Comparative transcriptomic analysis of DEGs related to phytohormones responding to stimuli, together with phytohormone-related transcription factors related to Aux/IAAs, MYBs and ERFs showed that the expression of most of the DEGs in the leaves of ‘QY10’ was lower than that of ‘W10’ (Figure 2A), while the opposite trend was observed in the root (Figure 2B). In general, the expression of Aux/IAAs was more abundant in the root of ‘QY10’ compared with ‘W10,’ while the expression of all ERFs was much higher in the leaf of ‘W10’ compared with ‘QY10’ (Figures 2A,B). The MYB family has been reported to be involved in the response to many phytohormones (Chen Y. H. et al., 2006; Lee et al., 2015). In this study, the expression of *MYB34* and *MYB47* in response to JA (Chen Y. H. et al., 2006) was significantly higher in both the root and leaves of ‘QY10’ compared with ‘W10’ (Figures 2A,B).

We further analyzed the expression profiling of the genes related to the phytohormone biosynthetic process and signaling pathway to demonstrate the utility of DGE for understanding the specific functions of these genes in the response of *B. napus* to B deficiency. The expression of the DEGs involved in the auxin-mediated signaling pathway in the leaves and root of ‘QY10’ was almost higher than those of ‘W10’ (Figures 2C,D). The expression of the DEGs involved in the ABA-mediated signaling pathway in the leaves of ‘W10’ was almost higher than those of ‘QY10’ (Figure 2E). The expression of the DEGs involved in the JAs biosynthetic process in the leaves of ‘QY10’ was lower than those of ‘W10’ (Figure 2F). In contrast to the JAs, the expression of the DEGs involved in the BRs biosynthetic process was higher in the leaves of ‘QY10’ compared with ‘W10’ (Figure 2G). The transcription profiling indicated that the expression of the phytohormone-related genes differed considerably between the B-tolerant and B-sensitive genotypes. To validate this difference, the concentrations of IAA, ABA, and JA, as well as its precursor OPDA or the conjugate JA-Ile, and the related gene expression were measured under B deficiency stress.

### Effect of B deficiency on IAA in ‘QY10’ and ‘W10’

**Figure 3:**
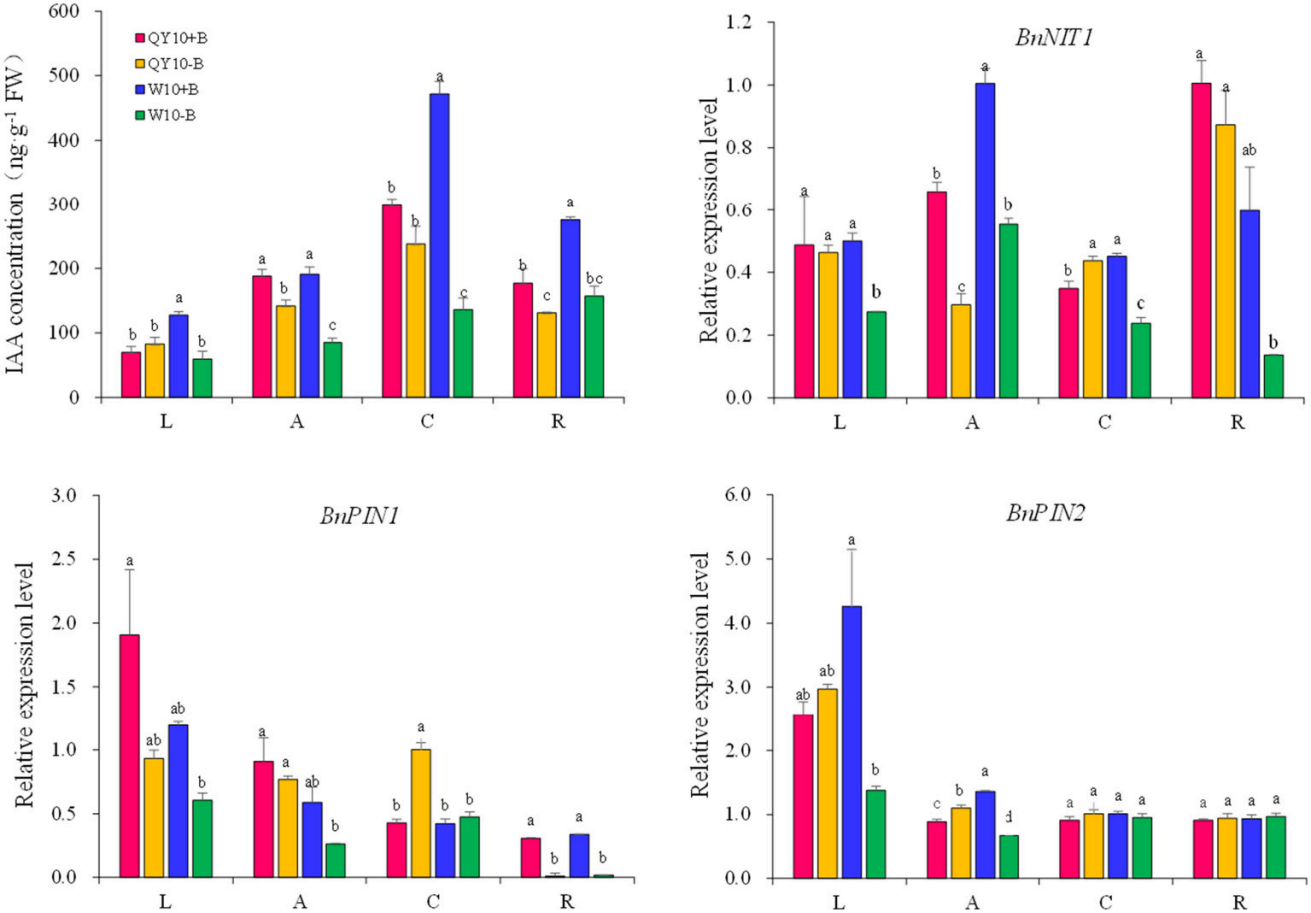
**IAA concentration and expression of IAA-related genes under long-term low-B stress**. *BnNIT1*: auxin biosynthesis-related gene. *BnPIN1* and *BnPIN2:* auxin efflux genes. Plants were grown hydroponically under sufficient B (+B, 25 μM B) or low-B (−B, 0.25 μM B) for 20 days. L, leaves; A, shoot apex; C, root crown; R, root. Values represent the mean ± SD of four independent replicates, and bars with different letters show significant differences for the same organ among the treatments (ANOVA, LSD, *p* < 0.05).

In terms of the long-term low-B stress, as shown in Figure 3, the IAA concentrations in all organs decreased compared with the sufficient B treatment, except in the leaves of ‘QY10.' The decline rate in the shoot apex, root crown and root of ‘QY10’ was 24.5, 20.3, and 26.0%, respectively. However, the rate was much more drastic in ‘W10,’ i.e., 55.4, 71.2, and 43.0%, respectively. Meanwhile, the IAA concentration in the leaves of ‘W10’ decreased by 53.5%. The changes in the expression of the IAA biosynthetic/transport-related genes were similar to the IAA concentration changes under long-term B deficiency. The IAA biosynthesis gene *BnNIT1* (NIT1, nitrilase1) was down-regulated by 55.0 and 44.9% in the shoot apexes and by 13.2 and 77.2% in the roots of ‘QY10’ and ‘W10,’ respectively. The auxin-efflux gene *BnPIN1* was down-regulated by 15.4 and 55.2% in the shoot apexes and by 96.7 and 94.5% in the roots of ‘QY10’ and ‘W10,’ respectively. Another auxin-efflux gene, *BnPIN2*, was down-regulated in leaves and the shoot apex, but only in ‘W10’ (Figure 3).

**Figure 4:**
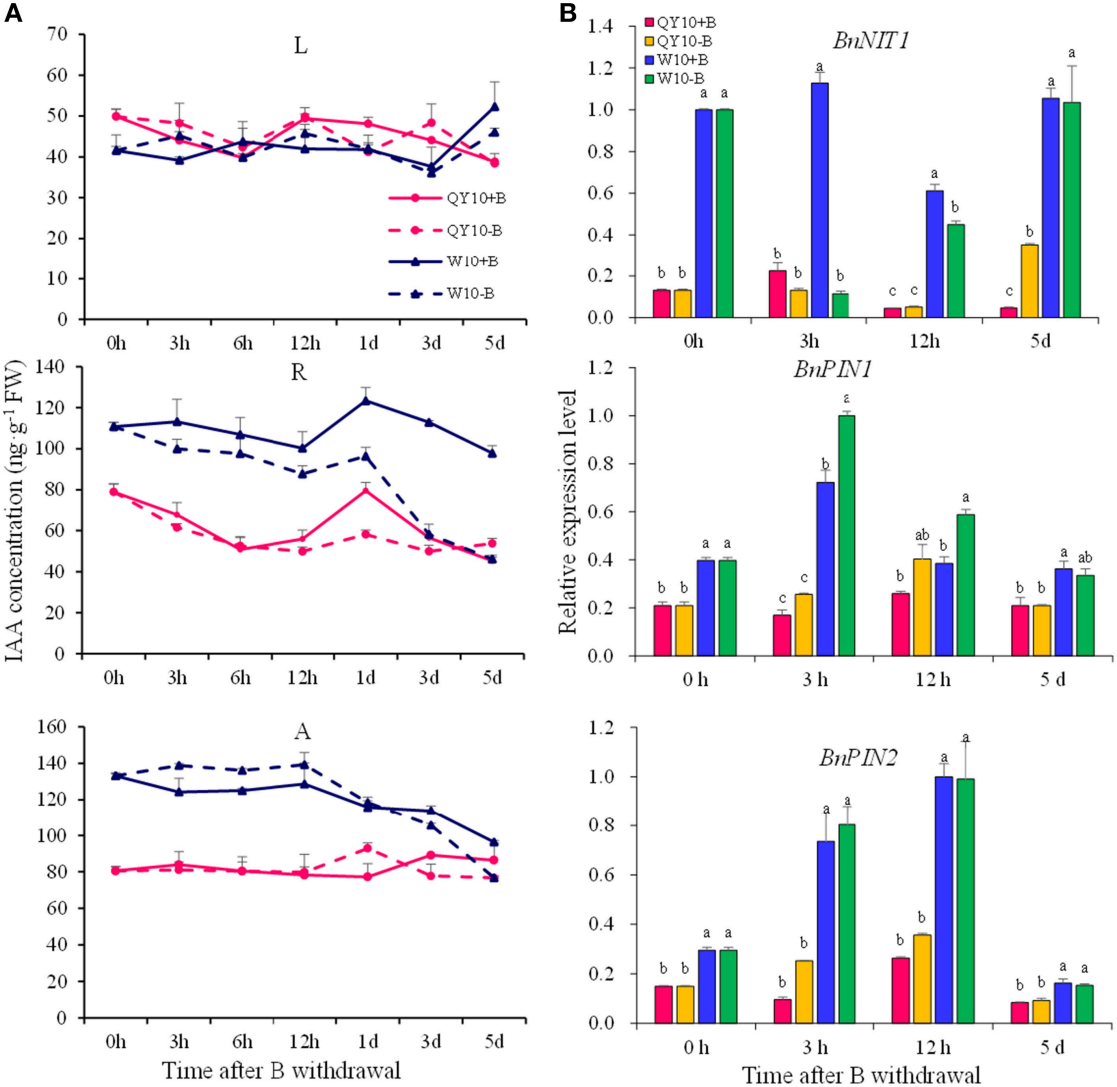
**IAA concentration and related-gene expression of ‘QY10’ and ‘W10’ under B-free starvation. (A)** IAA concentration. **(B)** IAA related-gene expression in root. After been grown hydroponically under sufficient B (+B, 25 μM B) for 12 days, half of the plants were transplanted to B-free solution (−B) and then sampled at different time points, and the other plants, which served as the control, were grown continuously with sufficient B. L, leaves; R, root; A, shoot apex. Values represent the mean ± SD of four independent replicates, and bars with different letters show significant differences for the same time among the treatments (ANOVA, LSD, *p* < 0.05).

As the removal of B could cause some early B-defective reactions (Goldbach et al., 2000), we carried out a short-term (5-day) B-free starvation experiment to corroborate the response of auxin to low-B. There was no obvious change in the IAA concentration in the three organs of the two genotypes within a 12-h period of B-free starvation compared with the B-sufficient condition. Compared with the B-sufficient condition, the IAA concentration in the shoot apex and the root of ‘W10’ decreased by 42.3 and 58.3%, respectively, from 0 h to the 5th day, while that of ‘QY10’ remained in a dynamic equilibrium (Figure 4A). These results demonstrated that IAA mediated B deficiency stress but did not instantaneously respond to B deficiency, which is in agreement with previous report by Xiong et al. (2001). Curiously, the expression of *BnNIT1* decreased at 3 and 12 h, especially in ‘W10,’ and then increased on the 5th day. The expression of *BnPIN1* was induced at 3 and 12 h, and then recovered to the level measured in the sufficient B treatment by the 5th day. The expression of *BnPIN2* was similar to that of *BnPIN1* (Figure 4B). These results pointed to the likelihood that the change in IAA in response to low-B stress was involved in the regulation of IAA biosynthetic/transport-related genes expression, which was induced by the B level in a complicated time-dependent way in *B. napus*.

In the long-term low-B stress and short-term B-free starvation experiments, ‘W10’ had a higher IAA concentration than ‘QY10’ under the B-sufficient condition (Figures 3, 4A). Meanwhile, the expression of the auxin biosynthesis gene *BnNIT1* was much higher in the root of ‘W10’ than in the root of ‘QY10,’ suggesting that ‘W10’ has a higher IAA demand (Figure 4B). This may partly account for the more severe symptoms exhibited by ‘W10’ under B deficiency.

### Activity of DR5:GFP and the PIN1::PIN1-GFP protein in *Arabidopsis* root

**Figure 5:**
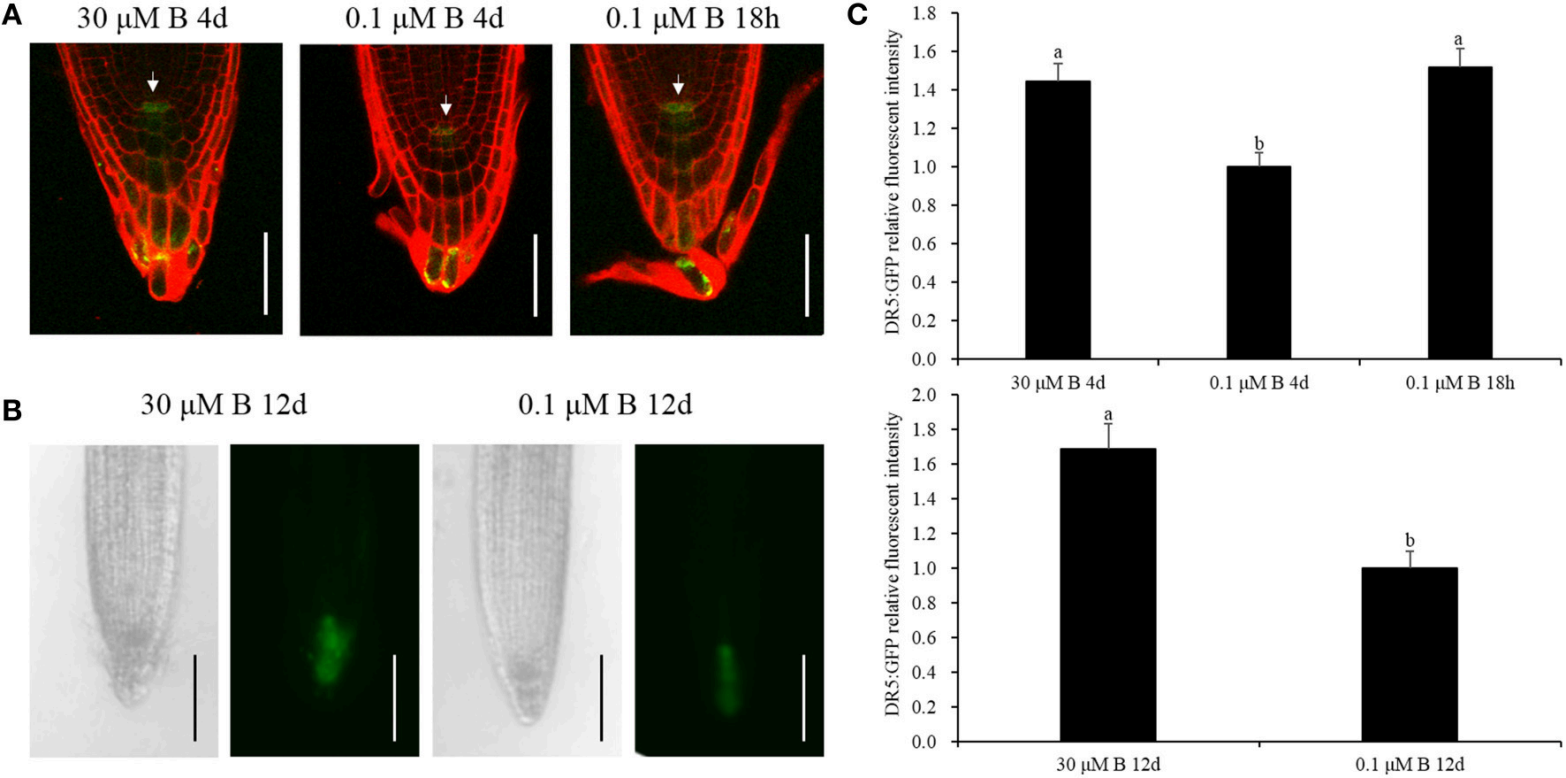
**GFP fluorescence of the DR5:GFP protein in the root tips. (A)** Transgenic *Arabidopsis thaliana* plants were grown in MS media containing 30 μM B for 4 days, 0.1 μM B for 4 days, or 30 μM B for 3 days and 6 h and then transferred to 0.1 μM B for 18 h. The cell wall was stained with PI (red). White arrows represent the quiescent center (QC). Scale bar = 50 μm. **(B)** Transgenic *Arabidopsis thaliana* plants were grown in MS media containing 30 or 0.1μM B for 12 days. Scale bar = 200 μm. **(C)** Quantification of the DR5: GFP fluorescence intensities in the quiescent center zones in **(A,B)**. Total of 10 seedlings were imaged for each treatment, and representative photos were shown. Values represent the mean ± SD of 10 independent replicates, and bars with different letters show significant differences (ANOVA, LSD, *p* < 0.05).

Auxin accumulation in the root mediated by auxin biosynthesis and transport is important for the onset of cell division in root meristems (Blilou et al., 2005; Zhu et al., 2015). Our assays afore-mentioned showed that low-B stress not only decreased auxin concentration but also changed its biosynthesis gene *BnNIT1* and the efflux gene *BnPIN1* mRNA abundance in the root in a time-dependent manner, and the decline was rather dramatic under long-term B deficiency. Thus, we tested *Arabidopsis* transgenic plants harboring DR5:GFP and PIN1::PIN1-GFP to investigate auxin distribution and flow in the root tip. Under the B-sufficient condition, DR5:GFP was mainly expressed in the root tips, specifically in the quiescent center (QC; Figure 5A), which is in accordance with previous report by Tanaka et al. (2006), and it was richly expressed when the seedlings were cultivated for 12 days (Figure 5B). The fluorescence intensity in the QC decreased to 69.2 and 59.2% after 4 and 12 days, respectively, under the low-B condition (Figures 5A,B). With respect to the 18-h B deficiency, there was no significant difference in the fluorescence intensity, except for a slight increase (Figures 5A,C). PIN1::PIN1-GFP fluorescence intensity decreased to 71.3% in the root subjected to low-B stress for 12 days, compared with that treated with sufficient B (Supplementary Figure 2), which implies that the relatively long-term B deficiency decreased the acropetal transport of auxin. These results indicate that auxin plays a vital role in root growth by decreasing the auxin concentration in the QC under B deficiency.

### Effect of exogenous IAA on B deficiency in ‘QY10’ and ‘W10’

**Figure 6:**
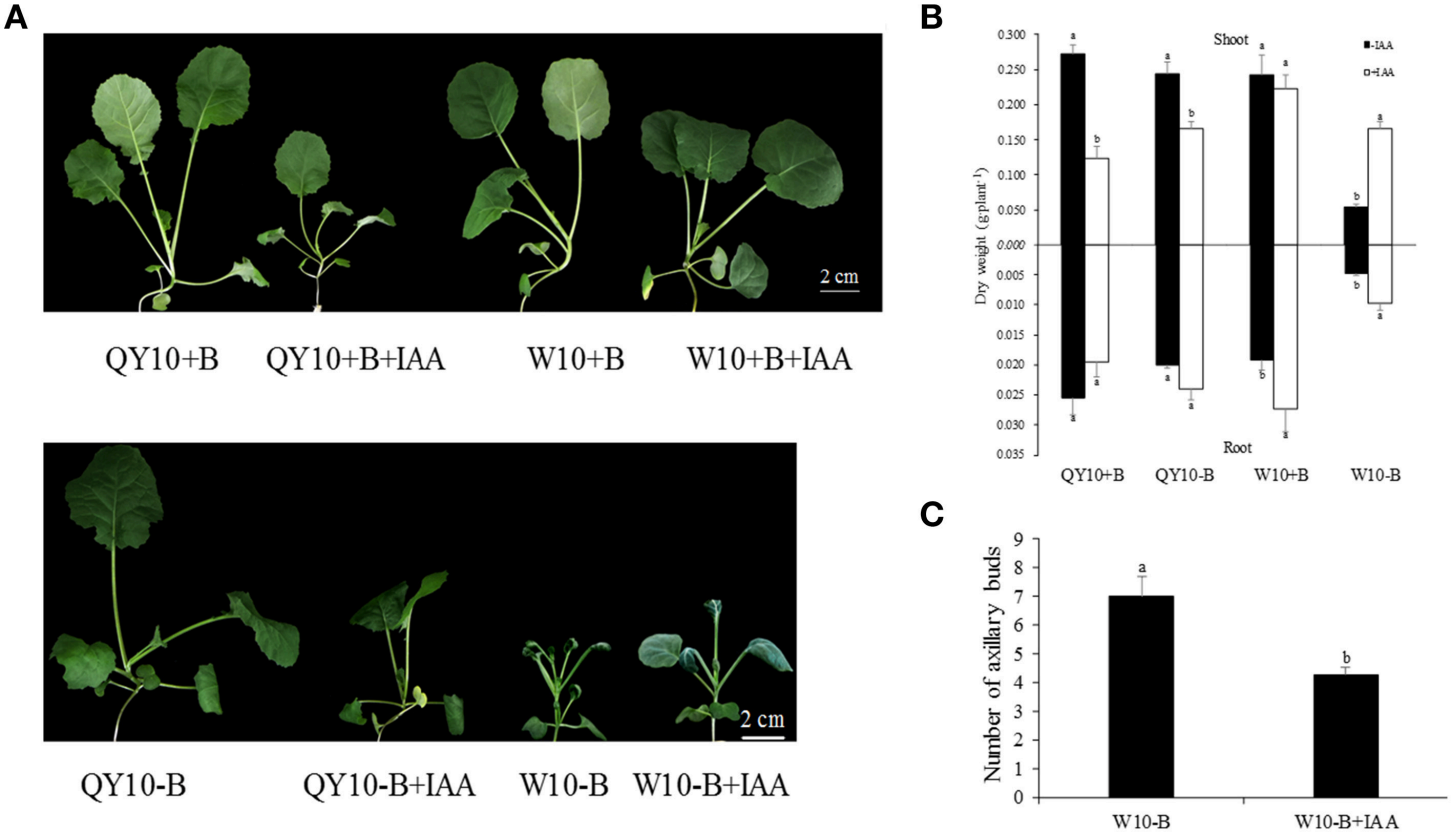
**Growth variations of ‘QY10’ and ‘W10’ under sufficient and low-B conditions with or without IAA. (A)** Phenotype, **(B)** dry weight, and **(C)** number of axillary buds of ‘QY10’ and ‘W10.’ Plants were grown hydroponically under sufficient B (+B, 25 μM B) or low-B (−B, 0.25 μM B) for 10 days, and then 10 μM IAA (+IAA) was supplied for another 10 days. Values represent the mean ± SD of four independent replicates, and bars with different letters show significant differences for the same organ and material between the treatments (ANOVA, LSD, *p* < 0.05).

The effects of exogenous IAA application on the ‘QY10’ and ‘W10’ phenotypes under sufficient- and low-B conditions are shown in Figure 6A. The shoot dry weight of IAA-treated ‘W10’ was significantly higher than that of ‘W10’ without exogenous IAA application (increased by 211.5%) under B deficiency. However, the shoot dry weights in IAA-treated B-deficient ‘QY10,’ B-sufficient ‘QY10’ and ‘W10’ were significantly lower compared with the plants without exogenous IAA application. The root dry weight increased except for ‘QY10’ under sufficient B. (Figure 6B). In addition, auxin significantly affected the number of axillary buds. There were an average of 4 axillary buds per plant in the QY10+B, QY10−B, and W10+B treatments (data not shown), compared with 7 under B deficiency (W10−B), which was reduced to 4.25 in the W10−B+IAA treatment (Figure 6C). The exogenous IAA experiment suggested that under B-sufficient conditions, plants with a suitable phytohormone status would suffer from a hyper-auxin toxicity when supplied with exogenous IAA. However, under B-deficient conditions, the B deficiency-sensitive genotype ‘W10’ with reduced IAA concentration was partly restored by exogenous IAA application.

### Effect of B deficiency on JAs in ‘QY10’ and ‘W10’

**Figure 7:**
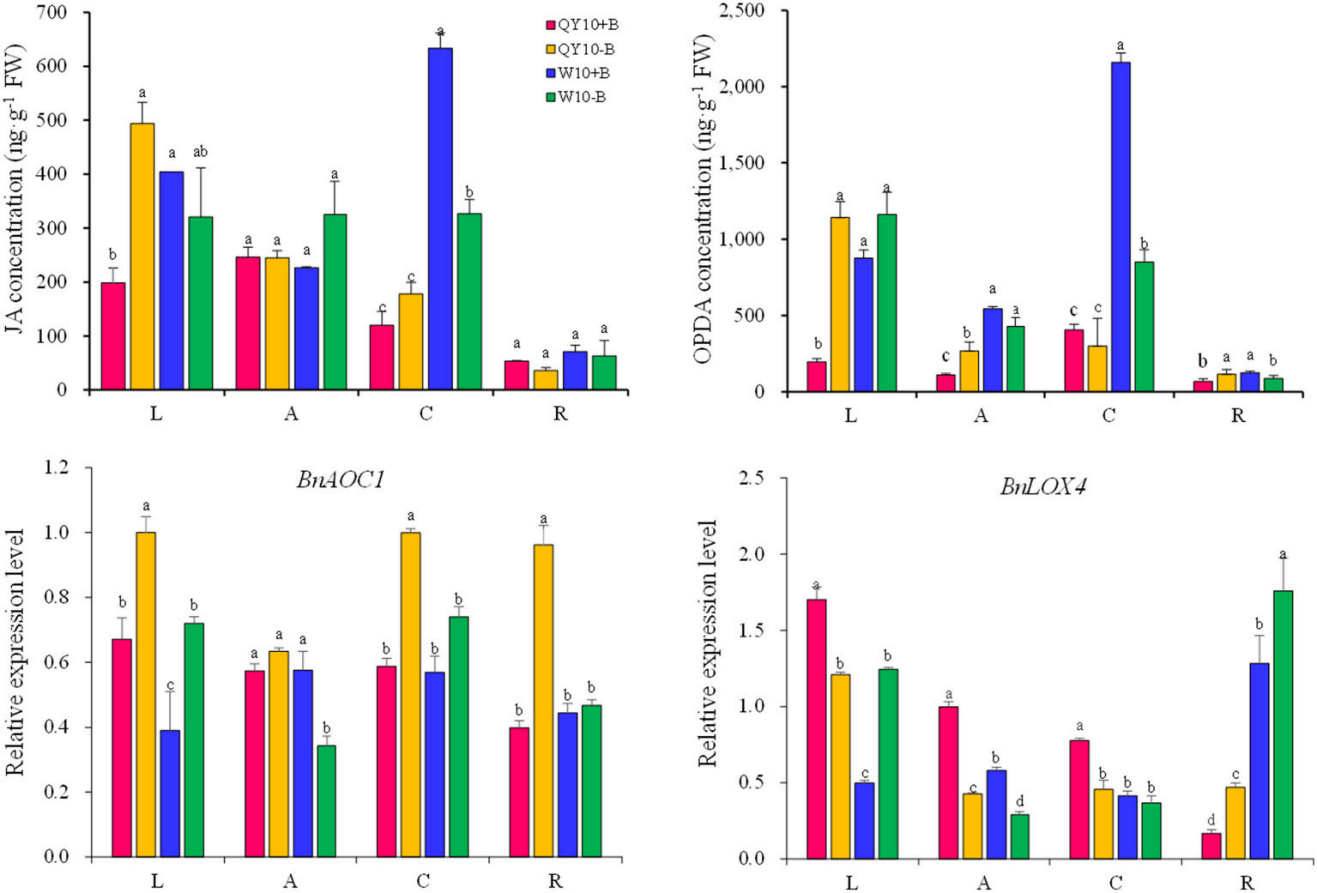
**JA and OPDA concentrations and expression of JAs biosynthesis genes under long-term low-B stress**. *BnAOC1, BnLOX4*: JA biosynthesis-related genes. Plants were grown hydroponically under sufficient B (+B, 25 μM B) or low-B (−B, 0.25 μM B) for 20 days. L, leaves; A, shoot apex; C, root crown; R, root. Values represent the mean ± SD of four independent replicates, and bars with different letters show significant differences for the same organ among the treatments (ANOVA, LSD, *p* < 0.05).

In this study, JAs were found to be the most abundant phytohormones among the three phytohormones tested in *B. napus*, especially in the leaves and root crown. Under long-term low-B conditions, the JA concentration in the leaves and the OPDA concentration in the leaves, shoot apex and root increased significantly in ‘QY10,’ whereas the JA concentration in the root crown and the OPDA concentration in the root crown and root of ‘W10’ decreased significantly (Figure 7). The expression of the JA biosynthesis genes *BnAOC1* and *BnLOX4* (AOC1, allene oxide cyclase1; LOX4, lipoxygenase 4) indicated an alteration trend under long-term B deficiency that was similar to the change in the JAs concentration in the leaves. Both *BnAOC1* and *BnLOX4* were up-regulated in the leaves of ‘W10’ under B deficiency, while the expression of *BnLOX4* in the leaves of ‘QY10’ was slightly down-regulated (Figure 7).

**Figure 8:**
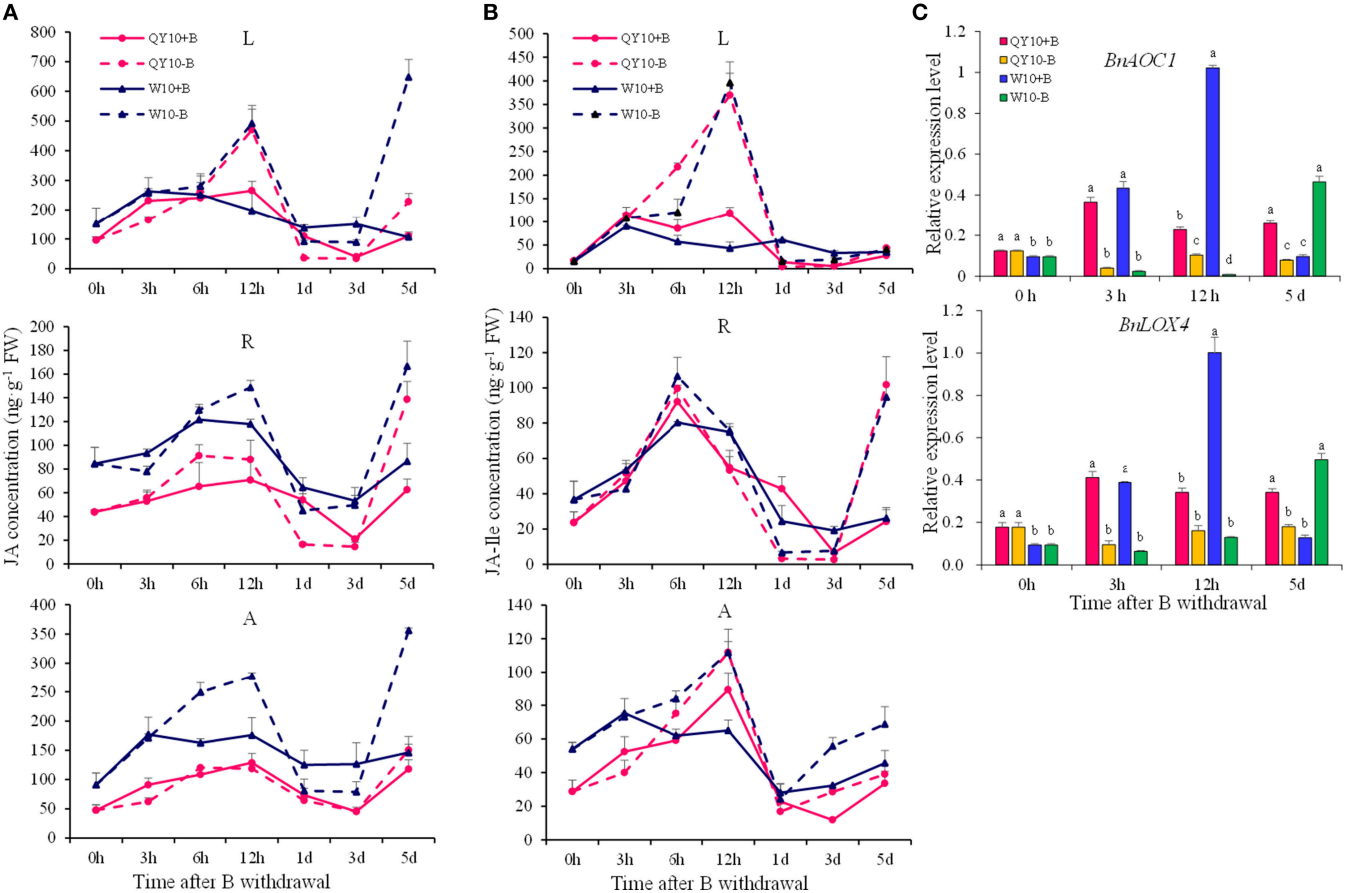
**JAs concentrations and related-gene expression of ‘QY10’ and ‘W10’ after been transferred to a B-free solution. (A)** JA concentration. **(B)** JA-Ile concentration. **(C)** Expression levels of the JAs biosynthesis genes *BnAOC1* and *BnLOX4* in the leaves of ‘QY10’ and ‘W10.’ After been grown hydroponically under sufficient B (+B, 25 μM B) for 12 days, half of the plants were transplanted to B-free solution (−B) and then sampled at different time points, and the other plants, which served as the control, were grown continuously with sufficient B. L, leaves; R, root; A, shoot apex. Values represent the mean ± SD of four independent replicates, and bars with different letters show significant differences for the same time among the treatments (ANOVA, LSD, *p* < 0.05).

Under short-term B-free starvation, an apparent pattern in the concentration of JAs was observed. For both ‘QY10’ and ‘W10,’ the JA concentration in the leaves, shoot apexes and roots peaked at 12 h (9:00 p.m.), followed by an attenuation at 1 day to a level that was parallel to that observed at 0 h (9:00 a.m.), regardless of whether the plants were under the B-sufficient condition or the B withdrawal treatment (Figures 8A,B). When the B-free starvation continued to the 5th day, the JAs concentration increased drastically. Compared with ‘QY10,’ the change in the JAs concentration under B-free starvation was more dramatic in the B-inefficient genotype ‘W10.’ On the 5th day, the respective JA concentration in the leaves, shoot apex and root increased by 107.2, 27.8, and 121.9% in ‘QY10’ compared with 507.1, 143.8, and 92.5% in ‘W10.’In addition, the respective JA-Ile concentration in the leaves, shoot apex and root increased by 58.1, 319.3, and 16.1% in ‘QY10’ compared with 15.1, 261.5, and 50.6% in ‘W10’ (Figures 8A,B). Compared with the B-sufficient condition, the expression of the *BnAOC1* and *BnLOX4* genes for JAs biosynthesis was down-regulated from 3 h to 5 days under short-term B-free starvation. However, these genes were significantly up-regulated on the 5th day in ‘W10’ (Figure 8C). Collectively, our data suggested that low-B stress increased the JA concentration in a time- and organ-specific manner.

### Effect of B deficiency on ABA in ‘QY10’ and ‘W10’

**Figure 9:**
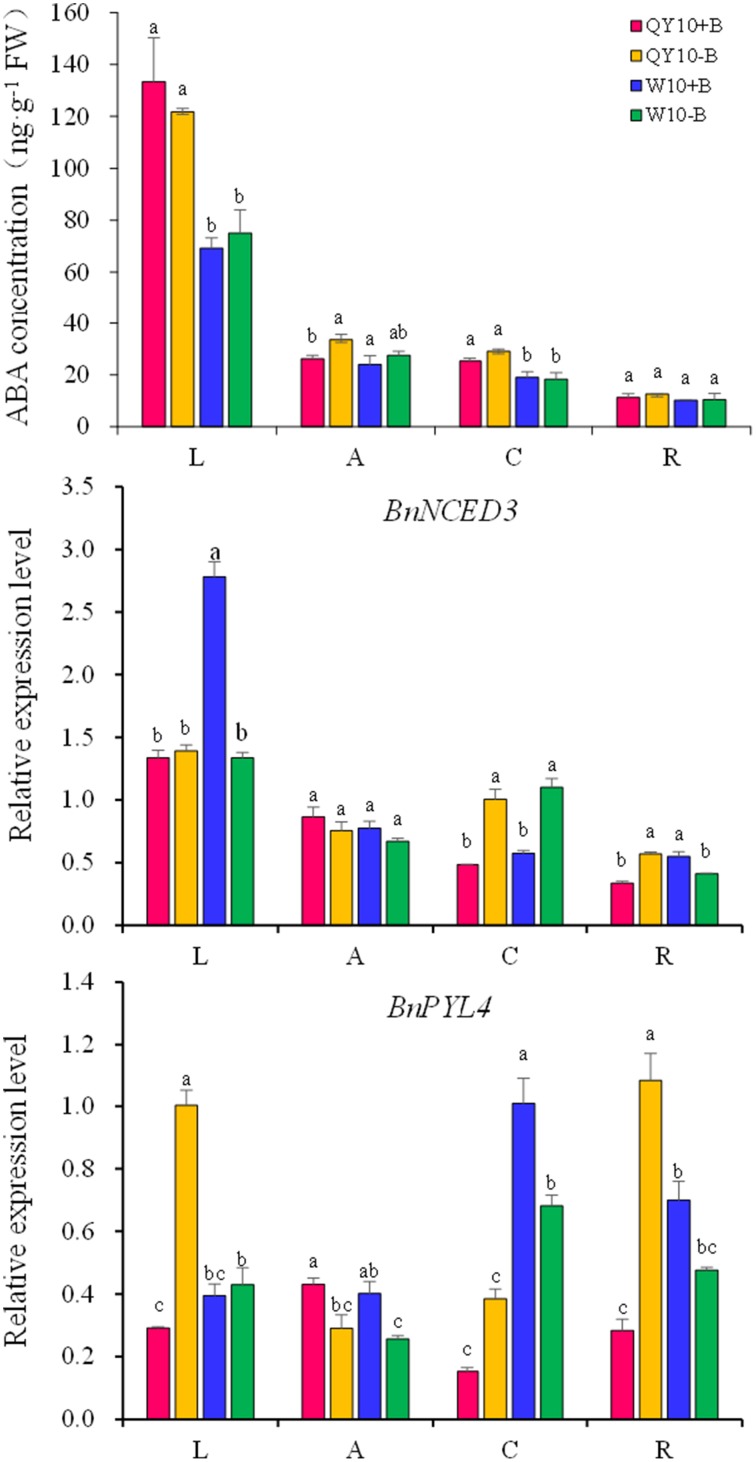
**ABA concentration and related-gene expression under long-term low-B stress**. *BnNCED3*, ABA biosynthesis-related gene; *BnPYL4*, ABA sensor gene. Plants were grown hydroponically under sufficient B (+B, 25 μM B) or low-B (−B, 0.25 μM B) for 20 days. L, leaves; A, shoot apex; C, root crown; R, root. Values represent the mean ± SD of four independent replicates, and bars with different letters show significant differences for the same organ among the treatments (ANOVA, LSD, *p* < 0.05).

The ABA concentration did not show significant changes under long-term low-B stress (Figure 9). While the expression of the ABA biosynthesis gene *BnNCED3* and the ABA sensor gene *BnPYL4* exhibited a rather strange alteration trend in ‘QY10’ and ‘W10’ for the whole plant. The expression of *BnNCED3* was up-regulated in the root crown in both ‘QY10’ and ‘W10,’ and the expression of *BnPYL4* was up-regulated in the leaves, root crown and root of ‘QY10.' However, in most cases, the expression of *BnNCED3* and *BnPYL4* was down-regulated in ‘W10,’ which may be attributed the disorder caused by long-term B deficiency in this genotype.

**Figure 10:**
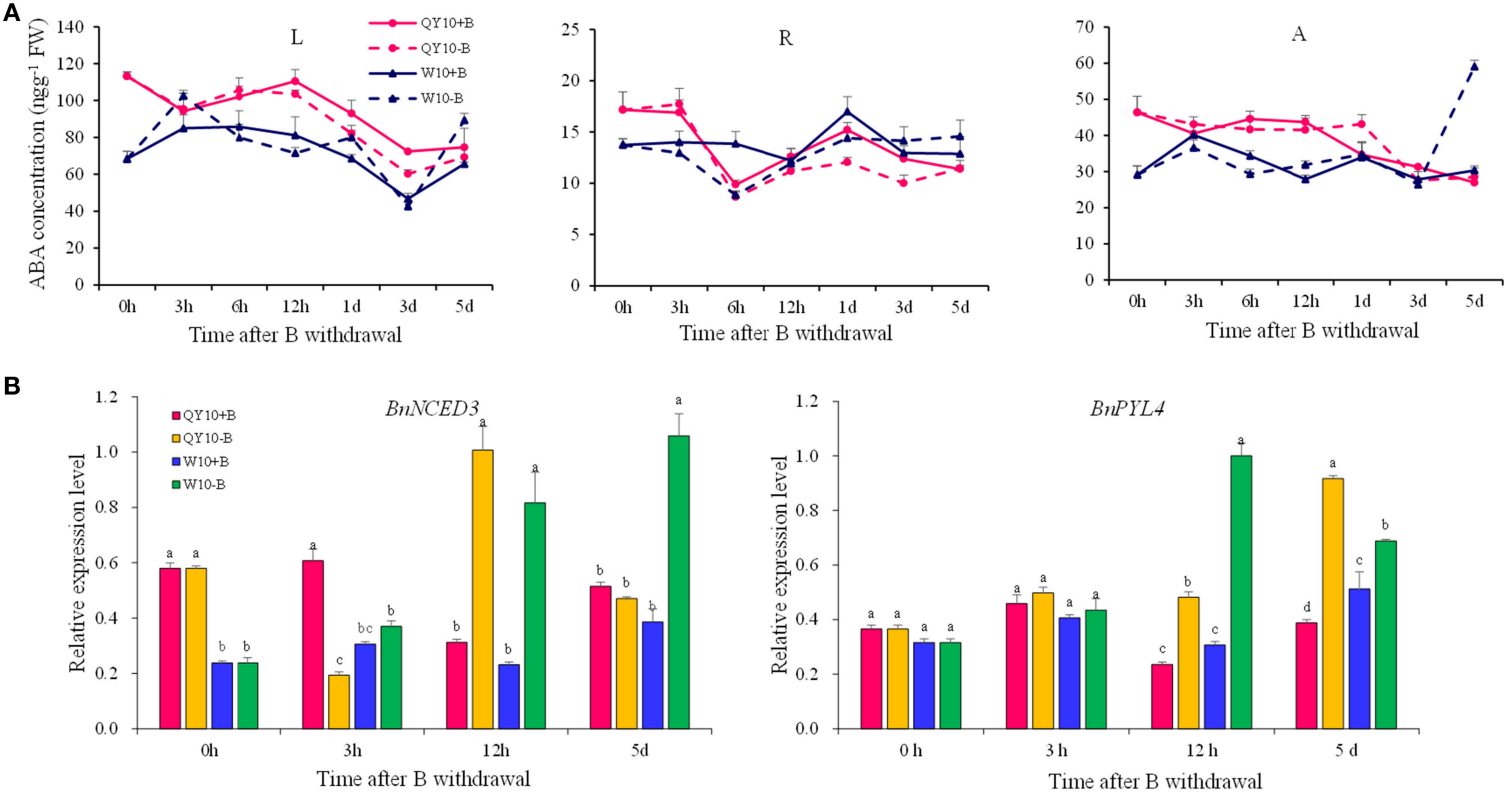
**ABA concentration and its biosynthesis gene expression in ‘QY10’ and ‘W10’ after been transferred to a B-free solution. (A)** ABA concentration. **(B)** ABA biosynthesis gene expression in the leaves of ‘QY10’ and ‘W10.’After been grown hydroponically under sufficient B (+B, 25 μM B) for 12 days, half of the plants were transplanted to B-free solution (−B) and then sampled at different time points, and the other plants, which served as the control, were grown continuously with sufficient B. L, leaves; R, root; A, shoot apex. Values represent the mean ± SD of four independent replicates, and bars with different letters show significant differences for the same time among the treatments (ANOVA, LSD, *p* < 0.05).

Under short-term B-free starvation, the ABA concentration remained in a dynamic balance except that it increased drastically from the 3rd day to the 5th day in the shoot of ‘W10’ (Figure 10A). *BnNCED3* and *BnPYL4* were up-regulated from 12 h to 5 days except for *BnNCED3* in ‘QY10’ on the 5th day, which confirmed the ABA concentration result (Figure 10B). These analyses suggested that the ABA concentration was more likely to be induced in the shoot of ‘W10’ under low-B conditions.

## Discussion

In the present study, analyses of phytohormone concentrations and related gene expression in different tissues of *B. napus* revealed for the first time that B deficiency exerted a considerable impact on IAA, Jas, and ABA homeostasis. In addition, the more disordered phytohormone status in the B-inefficient genotype ‘W10’ was attributed to its greater sensitivity to stress caused by B deficiency.

### Auxin-mediated axillary bud and root growth in response to B deficiency

In plants, the awe-inspiring number of processes is regulated by auxin and its directional cell-to-cell transport, e.g., the regulation of apical dominance (Phillips, 1975), promotion of the development of lateral roots (Casimiro et al., 2001), and the promotion of phototropism and gravitropism (Iino, 1995). Auxin homeostasis in plants is regulated by synthesis, catabolism, transport, and conjugation (Woodward and Bartel, 2005). Among the phytohormones, auxins are the most obviously involved with B nutrition of plants. Many B-defective symptoms exhibit considerable similarity to the symptoms of abnormal auxin concentrations, including the inhibition of root elongation, a change in the direction of cell expansion from longitudinal to radial, browning of root tips and proliferation of lateral roots (Pilbeam and Kirkby, 1983). However, auxin concentration changes in response to low-B stress have remained a dilemma for many years (Coke and Whittington, 1968; Crisp et al., 1976; Li et al., 2001; Wang et al., 2006). For the initiation of axillary buds, a minimum auxin accumulation in the leaf axil is necessary, and this auxin distribution is mediated by PIN1 (Wang et al., 2014). Auxin accumulation in the root apex is mediated by both auxin biosynthesis and transport (Grieneisen et al., 2007; Mano and Nemoto, 2012). In this research, it can be concluded that auxin concentration decreased under low-B (Figures 3, 4A), both in the shoot apex and root, which was consistent with previous studies (Li et al., 2001; Wang et al., 2006), and this may be attributed to the down-regulation of the auxin synthesis gene *BnNIT1* (Figures 3, 4B). Auxin concentration did not decrease until the 3rd day, indicating auxin concentration decrease was an indirect response, which was also consistent with previous studies (Li et al., 2001; Wang et al., 2006; González-Fontes et al., 2015). As auxin efflux genes, *PIN1* and *PIN2* play different physiological roles and represent different expression patterns in *Arabidopsis*. In the shoot, *PIN1* mainly regulates the auxin apical-to-basal flow (Tanaka et al., 2006). In the root, *PIN1* is expressed in the vascular bundle, charging auxin flow to the root tip to maintain root growth (Tanaka et al., 2006; Krecek et al., 2009). *PIN2* is mainly expressed in the epidermis and cortex in the root meristem, regulating meristem enlargement in the root (Tanaka et al., 2006; Kleine-Vehn and Friml, 2008). In the present study, the expression of *BnPIN1* in the shoot apex and root paralleled the IAA concentration under long-term B deficiency (Figure 3), which is in agreement with previous studies (Peng et al., 2012; Li et al., 2015). Meanwhile, the expression of *BnPIN2* declined in the root of ‘W10’ under long-term B deficiency, which is in agreement with Camacho-Cristobal et al. (2015) (Figure 3). The attenuated auxin transport may function synergistically with the reduced auxin biosynthesis to block auxin accumulation in low-B-treated roots. However, under short-term B-free starvation, the expression of *BnPIN1* was induced from 0 to 12 h, followed by a decrease in the root on the 5th day; *BnPIN2* exhibited a similar expression pattern (Figure 4B). Therefore, more attention should be paid to the different expression patterns of the *PIN* family in *B. napus* under long- and short-term B deficiency.

**Figure 11:**
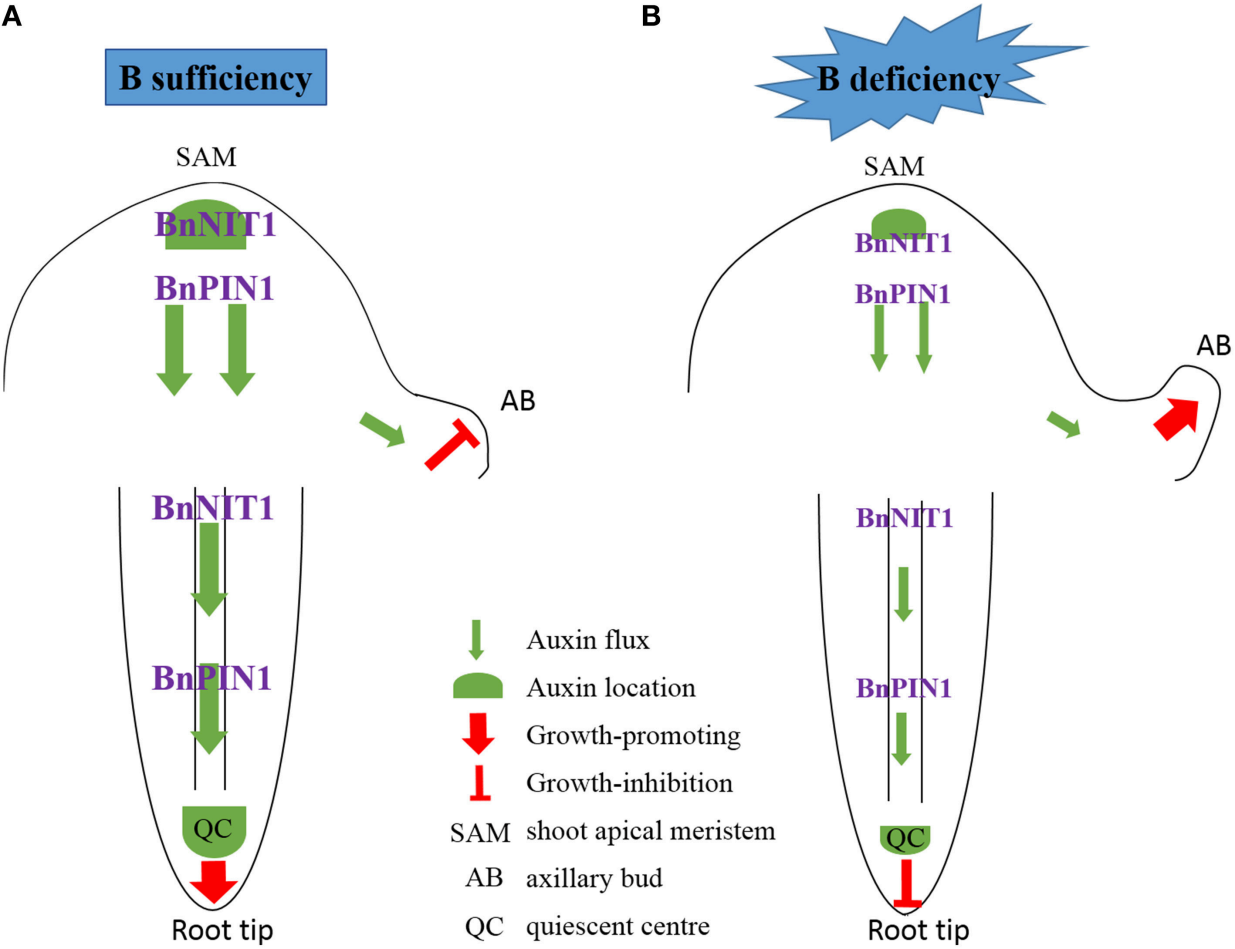
**Schematic model for auxin control of axillary bud and root tip growth in response to B deficiency**. Two situations were shown to illustrate the effect of auxin on AB and root growth, corresponding to plants supplied with sufficient or low-B. **(A)** Under B-sufficient conditions, plants are in a suitable auxin level, which slows down the outgrowth and elongation of the AB because of apical dominance. In the root, auxin was distributed mainly in the QC, which guarantees the growth of the primary root. **(B)** However, in the absence of **B**, the expression of *BnNIT1* and *BnPIN1* was down-regulated, which reduced the synthesis and basipetal transport of auxin in the shoot apex, thus preventing auxin accumulation in the AB, and initiating the outgrowth and elongation of AB. Meanwhile, low-B also reduced the synthesis and acropetal transport of auxin in the root, preventing auxin accumulation in the QC and inhibiting the growth of the primary root.

Combining the phenotypes of ‘QY10’ and ‘W10’ and the results of the IAA concentration, DGE analysis, quantitative gene expression analysis, *Arabidopsis* root DR5:GFP and PIN1::PIN1-GFP fluorescence observations and the IAA exogenous application experiment, we propose a model accounting for the specific effect of IAA on axillary bud (AB) and root growth under a relatively long-term B deficiency (Figure 11). Two situations were shown to illustrate the effect of auxin on AB and root growth, corresponding to plants supplied with sufficient or low-B. In many plant species, the outgrowth of the AB is inhibited by an active apical bud; this is, generally referred to as apical dominance (Wang et al., 2014). Under B-sufficient conditions, plants are in a suitable auxin level, which slows down the outgrowth and elongation of the AB because of apical dominance. In the root, auxin was distributed mainly in the QC, which guarantees the growth of the primary root (Figure 11A). However, in the absence of B, the expression of *BnNIT1* and *BnPIN1* was down-regulated, which reduced the synthesis and basipetal transport of auxin in the shoot apex, thus preventing auxin accumulation in the AB, and initiating the outgrowth and elongation of AB. Meanwhile, low-B also reduced the synthesis and acropetal transport of auxin in the root, preventing auxin accumulation in the QC and inhibiting the growth of the primary root (Figure 11B). We speculated a low-B tolerance mechanism based on phenotypic and phytohormonal evidences for ‘QY10’ and ‘W10,’ although the auxin changes in the two genotypes showed the same trends, there were significant differences when they were exposed to low-B stress. The magnitude of the auxin concentration reduction was much greater in ‘W10’ than in ‘QY10,’ and the decrease rate was more rapid (Figures 3, 4A). Meanwhile, auxin biosynthetic gene *BnNIT1* declined much more in each organ of ‘QY10’ than ‘W10’ (Figure 3). Transcription profiling proved that the expression of the DEGs involved in the auxin-mediated signaling pathway was almost higher in the leaves and root of ‘QY10’ compared with ‘W10’ under B deficiency (Figures 2C,D).

### Response of JAs to B deficiency

JAs are widely distributed in the plant kingdom and play multiple roles in physiological processes, such as inhibiting the growth of *Arabidopsis* seedlings (Staswick et al., 1992; Feys et al., 1994), regulating the resistance to insects and pathogens (Browse, 2009a) and the development of reproductive organs (Browse, 2009b; Wasternack et al., 2013). Whereas, the role of JAs in the resistance to mineral element deficiencies has been rarely reported. In the present study, the concentration changes of JA and its precursor OPDA or the conjugate JA-Ile were investigated in *B. napus* under low-B stress, together with the expression of JAs biosynthesis genes. Based on the above-mentioned analysis, JAs were found to be the most abundant phytohormone among the three phytohormones determined in *B. napus*. In addition, there was an obvious pattern in the concentration of JAs, which increased from morning (9:00 a.m.) to night (9:00 p.m.) and then declined by the next morning, i.e., the circadian rhythm, together with JAs biosynthesis gene expression (Figure 8). A similar phenomenon has been reported in rice (Liu et al., 2012). This may be caused by the self-activation of the JA biosynthesis mechanism (Sasaki et al., 2001), and the positive feedback mechanism of the JA biosynthetic enzymes (Creelman and Mullet, 1997). Under the long-term low-B condition, the alteration trend differed between the two genotypes: the JA concentration increased significantly in ‘QY10’ but decrease slightly in ‘W10.’ Interestingly, the JA concentration and its synthesis genes were up-regulated under low-B stress, especially in the leaves of ‘W10’ (Figure 7). It was likely that ‘W10’ plants were about to die after the 20-day B deficiency, the physiological function of these plants was seriously damaged and the cell biology activity was irreversibly inactivated (Oiwa et al., 2013), some responses measured were those which characterized plants that were severely damaged (Brown et al., 2002), so the JA concentration decreased. Under short-term B-free starvation, the JAs concentration increased on the 5th day and was more erratic in ‘W10’ (Figures 8A,B). However, the expression of JAs related genes was suppressed compared with the B-sufficient condition, except in ‘W10’ on the 5th day (Figure 8C). Therefore, the instant response of JAs to B deficiency may not be an increase, but rather a decrease, which requires further investigation.

The distinct responses of the JAs concentrations between the B-efficient and B-inefficient *B. napus* genotypes prompted us to determine whether JA interacted with B and if so, whether this interaction contributes to plant tolerance to B deficiency. In the DGE profiling analysis, the expression of all DEGs involved in the JAs biosynthetic process was lower in the leaves of ‘QY10’ than ‘W10’ under B deficiency (Figure 2F), further confirming the results of the measured JAs concentrations (Figures 7, 8A,B). Transcriptional regulation is one of the crucial mechanisms involved in protecting plants from various environmental stresses, and the key roles of many individual transcription regulators and their family classes in plant responses to environmental stresses have been well-demonstrated (Todaka et al., 2012). MYB21, MYB24, MYB34, MYB47, and MYB75 have been reported to be involved in the JA-mediated signaling pathway (Chen Y. H. et al., 2006; Qi et al., 2011). In the present study, the expression of MYB34 and MYB47 were significantly higher in both the root and leaves of ‘QY10’ compared with ‘W10’ (Figures 2A,B), indicating they may mediate the crosstalk between JA and B in *B. napus* genotypes and may open new avenues for future studies in this field.

### Response of ABA to B deficiency

Ever since its discovery, ABA has been intensively studied due to its versatile functions in plant development and stress physiology (Ye et al., 2012). The physiological roles of ABA were previously elucidated, including the establishment of seed dormancy, root development, stomatal closure, and so on (Cutler et al., 2010; Hirayama and Shinozaki, 2010). ABA is regarded as a phytohormone that can respond to nearly every type of stress (Chen J. et al., 2006; Ye et al., 2012). Wang and Zhou (1994) also reported that considerable amounts of ABA were generated enormously during every development period of cotton under B deficiency. In the present study, the expression of *BnNCED3* and *BnPYL4* was up-regulated over time (Figure 10B); on the 5th day, ‘W10’ underwent relatively severe B deficiency, and the ABA concentration increased drastically in the shoot of this genotype (Figure 10A). However, under long-term low-B stress, this difference was not apparent, and the ABA concentrations were similar to those measured in plants with sufficient B. A similar result was found in the root of wheat, which showed ABA only increased in the apical of root, not in the whole root under nutrient starvation (Vysotskaya et al., 2008). The expression of the DEGs involved in the ABA-mediated signaling pathway was almost higher in the leaves of ‘W10’ compared with ‘QY10’ (Figure 2G), and this is highly consistent with the result that the ABA concentration increased in the shoot of ‘W10’ but did not change in ‘QY10’ (Figure 10A). A similar alteration pattern was exhibited by the JAs and ABA, i.e., their concentrations increased in ‘QY10’ and decreased in ‘W10’ under long-term B deficiency, while their concentrations increased, especially in ‘W10,’ under short-term B-free starvation (Figures 7–10). The possible explanation for this is the same as that for the JAs, i.e., the leaf physiological function was seriously damaged in ‘W10,’ and the cell biology activity was irreversibly inactivated, leading to a decline in the phytohormone concentration in this genotype.

Although, our knowledge of the mechanism underlying the balance of various phytohormones in *B. napus* genotypes under B deficiency remains limited, these results indicate a dynamic and complex regulatory network that includes different phytohormonal synthesis/signaling responses to B deficiency in the molecular aspect. In contrast to previously published phytohormonal analyses in other plant species, we used two genotypes with contrasting tolerance to B deficiency, and formulated a comprehensive characterization of different transcriptional behaviors of phytohormones-related genes under B deficiency, so the finding is also potentially valuable for B efficiency identification and rapid nutrient diagnosis in the crop species susceptible to B deficiency.

## Author contributions

TZ and FX designed the experiments; TZ conceived the project, analyzed the data, and wrote the article with contributions of all the authors; YH, YPH, GD, and LS provided technical assistance to TZ; FX supervised and complemented the writing.

### Conflict of interest statement

The authors declare that the research was conducted in the absence of any commercial or financial relationships that could be construed as a potential conflict of interest.

## Abbreviations

AB: axillary bud
AOC1: allene oxide cyclase1
B: boron
BEC: B efficiency coefficient
BRs: brassinosteroids
*B. napus*: *Brassica napus*
CTK: cytokinin
DEGs: differentially expressed genes
DGE: digital gene expression
DM: dry matter
ETH: ethylene
FW: fresh weight
GA: gibberellin
GFP: green fluorescent protein
JAs: jasmonates
LOX4: lipoxygenase 4
NCED3: nine-cis-epoxycarotenoid dioxygenase 3
NIT1: nitrilase1
OPDA: JA biosynthetic precursor 12-oxophytodienoic
Pi: inorganic phosphate
PI: propidium iodide
PIN1: pin-formed 1
PIN2: pin-formed 2
PYL4: pyr1-like 4
QC: quiescent center
qRT-PCR: quantitative real time PCR
SA: salicylic acid
UFLC-ESI-MS: ultra-fast liquid chromatography-electrospray ionization tandem mass spectrometry.
