# Supplementary material for: Physiological and Transcriptional Analyses Reveal Differential Phytohormone Responses to Boron Deficiency in Brassica napus Genotypes
Source: Front Plant Sci. 2016 Feb 26;7:221. doi: 10.3389/fpls.2016.00221 (PMC4767905; doi:10.3389/fpls.2016.00221)
Supplement: Supplementary file 1 [file Table1.PDF]

Supplementary Table 1 Primer sequences used for quantitative RT-PCR

Gene	Primer sequences
BnNIT1	F-5′-AGATAAACTGGCGGAACTAGC-3′
	R-5′-CATGACCTTACGGTGCTTAC-3′
BnPIN1	F-5′-ACACCGACCCAATGCTCC-3′
	R-5′-GCCCATGACGAGGGTGTT-3′
BnPIN2	F-5′-TCGTCACGGCTACACCAATAG-3′
	R-5′-TCGCTCCTCTACCTCCTTTC-3′
BnAOC1	F-5′-TCTCGTCCCATTCACCAAC-3′
	R-5′-AAGATTCCAGTGCCACCAGT-3′
BnLOX4	F-5′-CATTGCCGATGTATGTGCC-3′
	R-5′-CTTGAATCCCTAACTTTAGAAGC-3′
BnNCED3	F-5′-CGACGGGCAGTTAGAATCCAC-3′
	R-5′-CGGCGAGAAGCGGAAGTA-3′
BnPYL4	F-5′-TCCGTCCAGGTCCCGATG-3′
	R-5′-CGGACCATACGGCAGAAAT-3′
Actin	F-5′- ACAGTGTCTGGATCGGTGGTTC -3′
	R-5′- TGCCTCATCATACTCAGCCTTG -3′
