# Supplementary material for: Physiological and Transcriptional Analyses Reveal Differential Phytohormone Responses to Boron Deficiency in Brassica napus Genotypes
Source: Front Plant Sci. 2016 Feb 26;7:221. doi: 10.3389/fpls.2016.00221 (PMC4767905; doi:10.3389/fpls.2016.00221)
Supplement: Supplementary file 2 [file Table2.PDF]

Supplementary Table 2 Total number of phytohormone-related genes identified using digital gene expression (DGE) profiling and differentially expressed genes (DEGs) ($P < 0.05$, $FDR < 0.05$) in 'QY10' and 'W10' in response to B deficiency and the proportion of DEGs accounting for the total number of genes.

		Root		Shoot	
	Total	DEGS	%	DEGS	%
auxin	979	186	19.00	256	26.15
ABA	486	96	19.75	145	29.84
ETH	369	80	21.68	106	28.73
BRs	203	37	18.23	62	30.54
JA	200	35	17.50	54	27.00
GA	199	26	13.07	35	17.59
CTK	157	27	17.20	38	24.20
SA	151	25	16.56	32	21.19
Total	2744	512	18.66	728	26.53
