# Supplementary figures and images for: Physiological and Transcriptional Analyses Reveal Differential Phytohormone Responses to Boron Deficiency in Brassica napus Genotypes
Source: Front Plant Sci. 2016 Feb 26;7:221. doi: 10.3389/fpls.2016.00221 (PMC4767905; doi:10.3389/fpls.2016.00221)

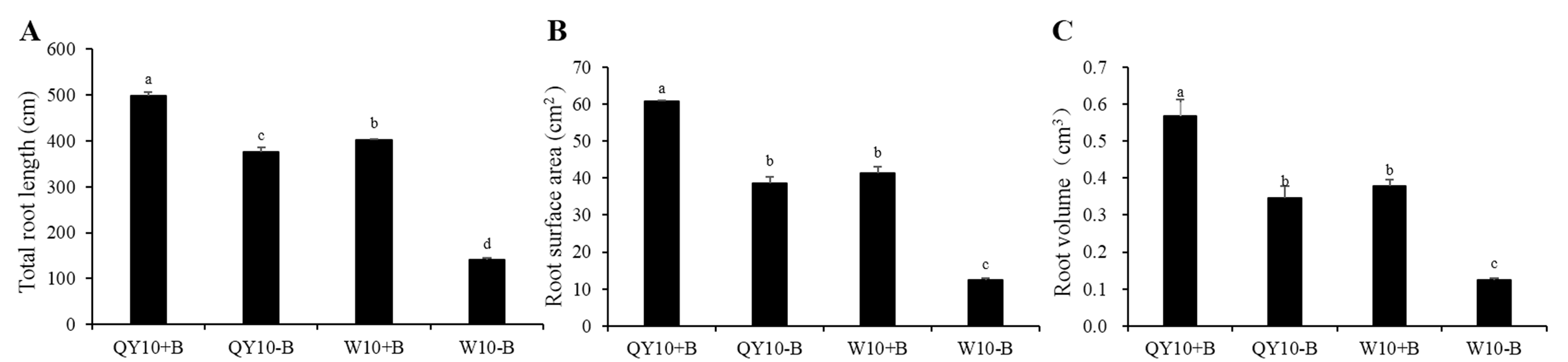

Supplement: Supplementary file 3 [file Image1.TIF]

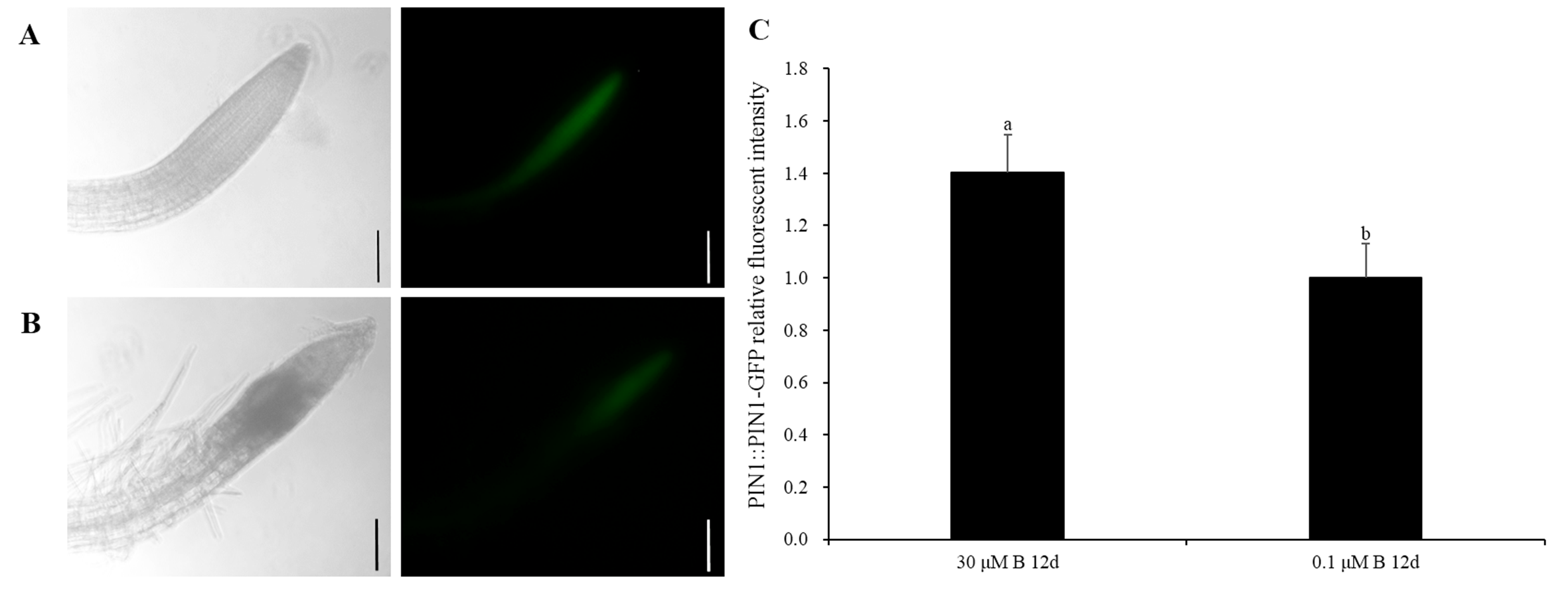

Supplement: Supplementary file 4 [file Image2.TIF]
